# Cold Atmospheric Plasma Enhances TGF-β1, CTGF Protein Expression, and Healing in Full-Thickness Skin Burns: An Animal Study

**DOI:** 10.3390/biom15070924

**Published:** 2025-06-24

**Authors:** Sahar M. Gebril, Fakhr El din M. Lasheen, Mohamed Khalaf, Amr Abdelhamed, Manal I. Bahkali, Fayez El Hossary, Mahmoud Rezk Abdelwahed Hussein

**Affiliations:** 1 Histology and Cell Biology Department, Faculty of Medicine, Sohag University, Sohag city 82524, Egypt; sahermohamed@med.sohag.edu.eg; 2 Zoology Department, Faculty of Science, Sohag University, Sohag city 82524, Egypt; flashein@yahoo.com; 3 Physics Department, Faculty of Science, Sohag University, Sohag city 82524, Egypt; mmmfoas@gmail.com (M.K.); fayez1004@hotmail.com (F.E.H.); 4 Department of Experimental Physics, Faculty of Mathematics, Physics, and Informatics, 842 48 Bratislava, Slovakia; 5 Dermatology, Venereology and Andrology Department, Faculty of Medicine, Sohag University, Sohag city 82524, Egypt; amr_abdelhamed@med.sohag.edu.eg; 6 Department of Pathology, Jazan Military Hospital, Jazan 82848, Saudi Arabia; 7 Department of Pathology, Faculty of Medicine Assiut University Hospitals, Assiut 71515, Egypt

**Keywords:** cold, atmospheric, plasma, wound, healing, acute, burn, growth, factors, immunohistochemistry

## Abstract

Cold atmospheric plasma (CAP) interacts with tissues, leading to fast wound disinfection. Given the frequent global burden of burn injuries and the risks of infection associated with acute full-thickness burns (FTBs), this investigation examined CAP as a potential therapeutic method for wound healing due to its antimicrobial and pro-healing effects. Here, we examined the impacts of CAP on the healing of wounds resulting from acute FTSBs. We established an animal model that included four groups: (1) healthy control animals without burns, (2) untreated animals with acute FTSBs, (3) animals with acute FTSBs treated with CAP for 5 s per day for 21 days, and (4) animals with acute FTSBs treated with CAP for 10 s per day for 21 days. Wound healing was assessed using immunohistological methods. In animals with FTSBs, CAP therapy was accompanied by (i) accelerated wound closure, (ii) enhanced regeneration of the dermis and epidermis, and (iii) increased protein expression of transforming growth factor-β1 (TGF-β1) and connective tissue growth factor (CTGF). These changes were more pronounced following CAP treatment for 10 s per day compared to CAP treatment for 5 s per day.

## 1. Introduction

Burn injuries impact more than six million individuals globally each year, highlighting a significant public health concern [1]. Among these, skin burns are particularly common and can be devastating, whether occurring in civilian contexts or during military operations [2]. Common causes of skin burns include exposure to fires, flames, heated objects, and various chemicals [3].

Despite advances in the treatment of skin burns, infections remain a critical issue, accounting for 42% to 65% of burns-related deaths. This high mortality rate is largely due to the emergence of antibiotic-resistant bacteria, which complicates wound healing and underscores the need for developing novel treatment strategies [4].

Third-degree burns, also referred to as full-thickness skin burns (FTSBs), penetrate through the entire skin layer into the underlying subcutaneous tissues. These injuries often appear white or charred and are typically insensate due to nerve damage. Healing of these severe injuries occurs through wound contracture, and skin grafts are frequently required. Patients with acute FTSBs require specialized medical attention and often hospitalization for comprehensive management [5].

Cold atmospheric plasma (CAP) is an ionized gas containing a high density of charged particles, including electrons and ions [6]. In addition to these charged components, CAP is composed of ultraviolet photons and generates various reactive species, such as oxygen-free radicals and nitrogen-based compounds [7]. Owing to its strong antimicrobial properties, the application of CAP is regarded as one of the most potent sterilization techniques, effectively eliminating microorganisms upon contact. Moreover, CAP therapy can diminish bacterial populations in injured tissues, thereby promoting wound healing [8].

Several growth factors are important for the wound healing process, particularly transforming growth factor-beta (TGF-β1) and connective tissue growth factor (CTGF) [9,10]. TGF-β1 functions as a pleiotropic and anti-inflammatory cytokine. It orchestrates wound healing by facilitating keratinocyte proliferation and migration, enhancing extracellular matrix synthesis, and regulating immune responses [10]. CTGF is a secreted protein associated with the extracellular matrix. It functions as a downstream mediator of TGF-β1 [9,11], amplifying its effects and interacting with various cytokines and growth factors. These include tumor necrosis factor (TNF), Interleukin-6 (IL-6), monocyte chemoattractant protein-1 (MCP-1), basic fibroblast growth factor (bFGF), and vascular endothelial growth factor (VEGF)—to further support tissue repair and regeneration [12].

Research has highlighted the critical roles of TGF-β1 and CTGF in various phases of wound healing, including fibroblast activation, angiogenesis, and fibrosis [13,14,15]. For example, Tarafder et al. used an explant model to examine the impact of CTGF and TGF-β3 on the healing of avascular meniscus tears. Their findings showed that a high dose of CTGF combined with a slow sustained release of TGF-β3 markedly enhanced wound healing [16].

CTGF overexpression is observed in wound healing and several fibrotic conditions. In a study using rabbit models, Sisco et al. highlighted the role of CTGF in dermal wound healing and scar formation, reporting elevated CTGF mRNA levels in hypertrophic scars [13]. Treatment with antisense oligonucleotides targeting CTGF led to a significant decrease in myofibroblast numbers within scars, alongside reduced transcription of inhibitors of the matrix metalloproteinases (TIMP-1) and collagen types I and III [13].

Currently, the beneficial effects of CAP on the healing of acute FTSBs are not yet fully understood, highlighting a pressing need for research to explore this topic further. To address this gap, we developed a guinea pig model to examine the effects of CAP on wound healing.

## 2. Materials and Methods

This research study was approved by the Ethics Review Committee of the Faculty of Medicine at Sohag University, which included authorization for animal care and experimentation, under approval number Sohag-IACUC/IRB:222031.

Materials and reagents: The floating electrode dielectric barrier discharge plasma device and instrumentation (FE-DBD) was developed using a high-voltage power supply operating at 50 kHz. This system incorporated a stainless-steel electrode, measuring 1 mm in thickness and covering an area of 3 cm^2^. To ensure electrical isolation from the ground, a polymer plate, also 1 mm thick, was employed.

The dissipated plasma power was calculated from the Q-V relationship using the Lissajous method and was adjusted to 0.5 W. A filamentary discharge was observed after applying high voltage, as shown in the current–voltage waveforms. Optical emission spectroscopy (OES) revealed that the dominant emission spectra correspond to the second positive nitrogen system [17]. Power measurements, current–voltage waveforms, and optical spectra were recorded following established procedures used by other research groups. A summary of the reagents used in this study is shown in Table 1.

The rationale behind performing an acute FTSB model using guinea pigs was as follows: Several factors justify the use of guinea pigs in our current model of acute FTSBs. Importantly, the physiological and anatomical characteristics of guinea pig skin closely resemble those of human skin, as indicated by previous studies [18,19]. For example, guinea pig skin is devoid of the extensive panniculus carnosus found in small “loose” skinny animals, which may influence the healing process [20]. Furthermore, the expression patterns of several growth factors, including TGF-β1, and CTGF display similar regulatory patterns in both humans and pigs during the wound healing process [20]. Collectively, these factors underscore the suitability of guinea pig skin as a reliable model for investigating acute FTSBs with translational relevance to human health [18,21].

Study groups: We utilized a cohort of 24 guinea pigs, aged from 4 to 5 months and weighing approximately 400 g ± 50 g. The animals were obtained from the Animal Facility of the National Research Centre in Cairo, Egypt. The handling, care, and welfare of the animals conformed to the guidelines established in the *Guide for the Care and Use of Laboratory Animals* by the National Research Council. Before the experimental procedures, the animals were appropriately housed and underwent a one-week acclimatization period, during which they were provided with standard guinea pig feed and had unrestricted access to water. The environmental conditions were maintained with a 12 h light/dark cycle, a temperature of 25 ± 2 °C, and humidity levels of 55 ± 5%.

Following acclimatization, the animals were randomly assigned to four experimental groups (n = 6 per group). Group I comprised healthy controls with no induced FTSBs. Group II included animals with acute FTSBs that received no treatment. Group III consisted of animals with acute FTSBs treated with topical CAP for 5 s daily over a period of 21 days. Group IV received topical CAP treatment for 10 s daily for the same duration.

Precise control of the CAP treatment parameters, particularly exposure time and power input, is critical for maximizing therapeutic efficacy while minimizing off-target effects. Power input directly influences the energy density of the plasma, thereby impacting its biological activity. Consequently, both treatment duration and power must be carefully optimized. Fridman et al. demonstrated that plasma at 0.8 ± 0.2 W achieved skin sterilization in 3 ± 1 s, whereas treatment at half that power required 10 ± 4 s to achieve a similar effect [17]. In our previous work, we found that FE-DBD treatment at 0.5 W for 10 s enhanced diabetic wound healing, while extending the exposure to 20 s produced detrimental effects [17,22,23]. Based on these findings, the present study investigates the therapeutic efficacy of shorter treatment durations (5 and 10 s) to further refine CAP application parameters. To summarize, CAP was applied to wounds for brief durations—typically 5 to 10 s—to balance therapeutic efficacy while minimizing the risks of cytotoxicity and other side effects of cytotoxicity and other side effects. Previous studies lend support to this strategy [24,25]. All experimental procedures were carried out in the Animal House of the Faculty of Science at Sohag University. A summary of the study groups is presented in Table 2.

Induction of acute FTSBs: Following the acclimatization period, the dorsal skin of each animal was prepared by shaving the area using electric clippers. The skin was thoroughly cleansed with soap and water and subsequently treated with 70% isopropyl alcohol to ensure aseptic conditions [26]. To induce anesthesia, the animals were administered Xylazine at a dosage of 10 mg/kg and Ketamine at 75 mg/kg via intramuscular injection [20].

We induced acute FTSBs on the dorsal region using a standardized contact burn model, as described by previous studies [27,28,29]. A metal bar with a surface area of 40 mm^2^ maintained at a contact temperature of 170 °C, was utilized for this purpose. The temperature of the bar was accurately measured using a digital infrared thermometer (TP9, TROTEC).

To induce acute FTSBs, the preheated end of the metal bar was preheated to 170 °C and was applied to the prepared skin surface for 10 s under its weight, without additional pressure. A 5 min interval was maintained between successive applications to allow the bar to return to the target temperature.

Throughout the study, all animals with induced acute FTSBs were housed individually in separate cages. They were provided with a regular diet devoid of antibiotics and had ad libitum access to water. On day 21 post-burn, all animals were humanely euthanized. The skin specimens with induced acute FTSBs were excised from the burn sites, alongside specimens from healthy control skin. The tissues were fixed in a neutral buffered solution of 10% for a period of 72 h before undergoing histological processing (Tissue processor, Leica TP1020 Benchtop, Germany).

Morphological evaluation of the skins with acute FTSBs: We used a digital camera to capture images of wound healing and closure. At designated time points, the diameters of the wound sites were measured to determine the percentage of wound healing. The formula used for calculating the percentage of wound closure was as follows:

% Wound closure = [(Wound area _0_ − Wound area _21_)/Wound area _0_)] ∗ 100

Histological evaluation of the skins with acute FTSBs: For the histological assessment of skin specimens affected by acute FTSBs, 4 μm thick paraffin-embedded sections were prepared and stained with Hematoxylin and Eosin stain (H&E) to evaluate the histological changes. We used Orcein and Masson’s trichrome stains to assess the dermal elastic and collagen fibers, respectively [30]. To investigate the composition of glycogen and proteoglycans in the epidermal basement membrane, Periodic Acid-Schiff (PAS) staining was performed, following standardized protocols [31,32]. All staining reagents were purchased from Sigma-Aldrich Chemical Co., Ltd. (St. Louis, MO, United States).

Immunohistochemical evaluation of TGF-β1, CTGF, and Ki-67 protein expression: Immunohistochemical staining was performed in accordance with the methodologies established by other researchers [33]. After the deparaffinization process, the rehydrated tissue sections underwent heat-induced epitope retrieval using a citric acid buffer at pH 6.0. Subsequently, a 3% endogenous blocking agent was applied to block endogenous peroxidase activity. To further reduce non-specific binding, 10% normal goat serum was utilized. The sections were then incubated overnight at 4 °C with primary antibodies specific to TGF-β1, CTGF, and Ki-67, followed by the application of secondary antibodies for detection. We used a DAB chromogen signal detection kit (refer to Table 1). Finally, we counterstained the sections using Mayer’s Hematoxylin and examined them under a light microscope (OLYMPUS BX50, Olympus Corporation, Japan).

Negative controls: The negative controls were established by excluding the primary antibody and substituting it with a phosphate-buffered saline solution.

Histomorphometric analysis: We used a Leica Dm 3000 microscope (Leica, Germany) in conjunction with the Leica LAS EZ software suite (LAS EZ V 3.4.0, Germany) to scan and capture images of the stained skin sections. Morphometric analysis of collagen and elastic fibers was conducted utilizing Orcein and Mallory’s trichrome stains, respectively. The percentage of stained dermal area was quantified across 18 randomly selected high-power fields at 400× magnification, following established protocols [33].

In addition, we assessed the expression of TGF-β1 and CTGF, as well as the number of Ki-67-positive cells, in both the dermis and epidermis using J analyzer software (IMAGE J/FIJI 1.46r, National Institutes of Health) [34]. The results are presented as the mean percentage of the stained area ± standard deviation (±SD).

Statistical analysis: We performed group comparisons using analysis of variance (ANOVA) via the SPSS statistical package for Windows, version 16.0. Statistical significance was established at *p* < 0.05, with results expressed as mean ± SD for each group, based on assessments by two independent observers.

## 3. Results

**Figure 1:**
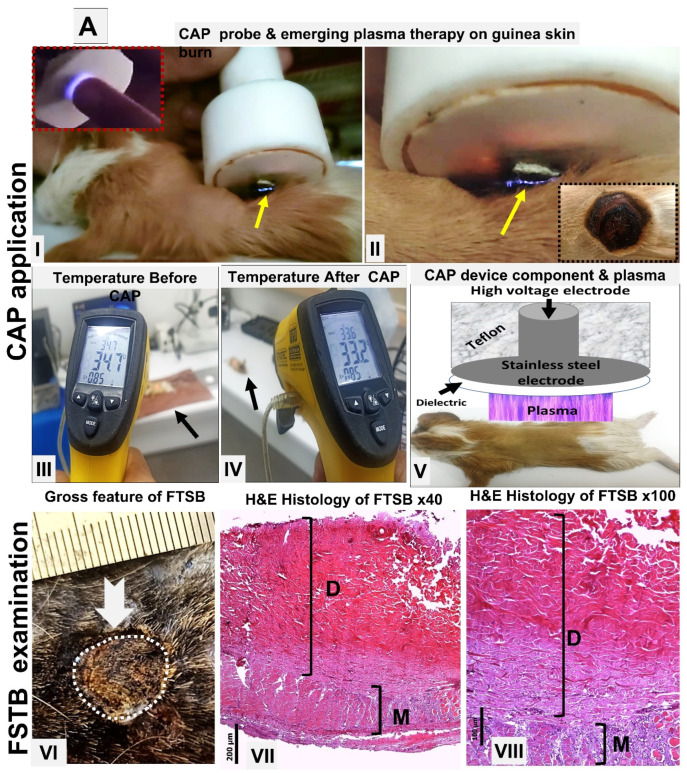

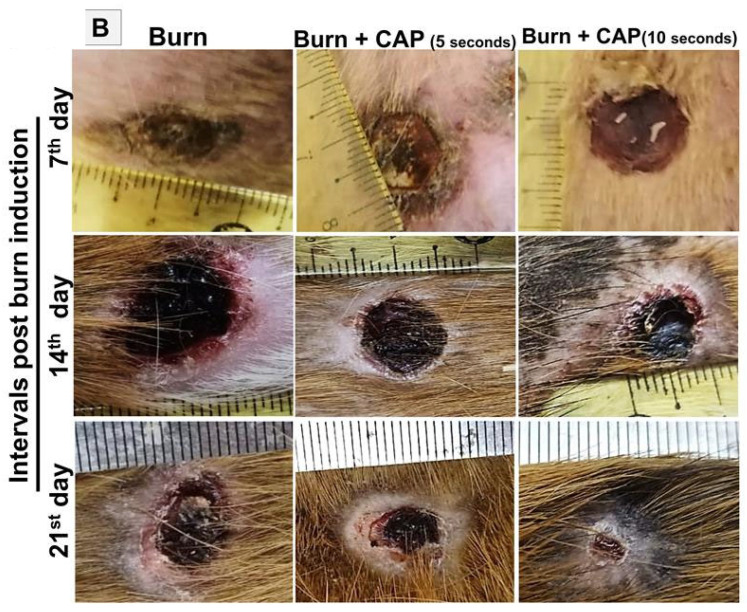
(**A**) Acute FTSBs in guinea pigs. (**A**) Overview of CAP treatment setup and application. (**A**: **I**–**II**) Application of the CAP device probe to a burn site on guinea pig skin. The emerging plasma (indicated by a yellow arrow) is visible at the treatment site. Inset: The plasma discharge appears as a visible flash of light on a human fingertip, demonstrating its visual characteristics. (**A**: **III**–**IV**) Skin temperature measurements on shaved dorsal skin of control guinea pigs before and after CAP treatment. A minimal decrease from 34.7 °C to 33.2 °C confirms the non-thermal nature of the plasma. (**A**: **V**) Schematic diagram of the CAP device structure and its positioning during treatment on the skin of the experimental animal. (**A**: **VI**–**VIII**) Acute FTSB (thick white arrow) of the skin of the back of the guinea pig with the destruction of the epidermis, and dermis denaturation (D) extending into the muscle layer (M) (H and E stains, magnifications: ×40 and ×100 for (**A-VII**) and (**A-VIII**), respectively. Scale bars: 200 µm (**A-VII**) and 100 µm (**A-VIII**), respectively). (**B**) Time interval macroscopic follow-up burns in guinea pigs. Gross evaluation of acute full-thickness skin burns on the guinea pig’s back at 7, 14, and 21 days post-induction across different groups.

**Figure 2:**
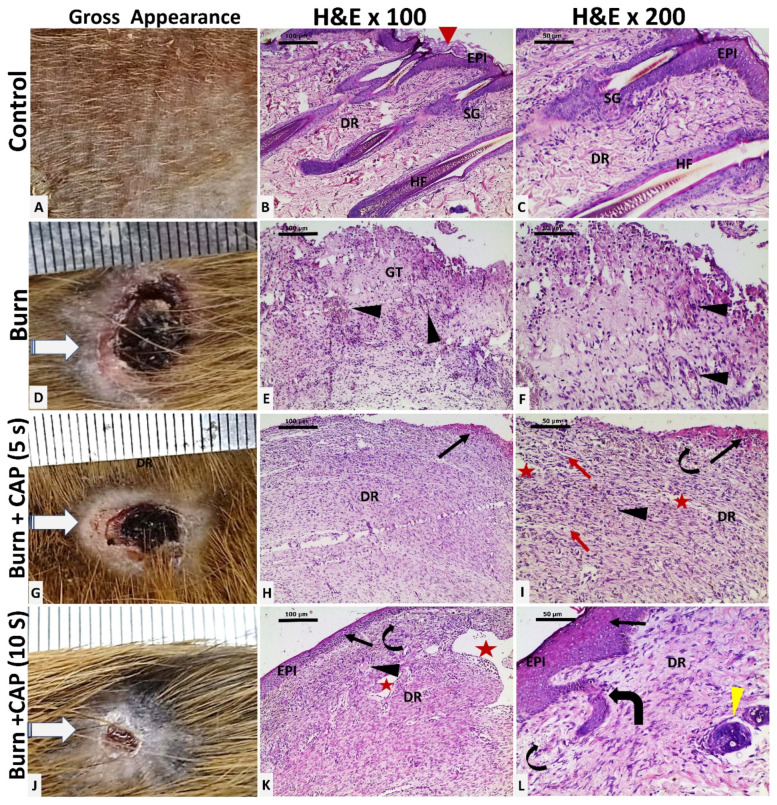
Histological evaluation of guinea pig skin following acute FTSBs. (**A**–**C**) (Group I—Control): The shaved back skin of the guinea pig appeared normal. Histology showed stratified squamous epithelium (Keratin: red arrowhead) in the epidermis (EPI), with hair follicles (HF) and sebaceous glands (SG) in the dermis (DR), among loose (papillary) and dense (reticular) collagen fibers (Magnifications: (**B**)—×100; (**C**)—×200). (**D**–**F**) (Group II—Untreated acute FTSBs): Grossly, the burn site was covered by crusts (**D**). Complete skin destruction is replaced by granulation tissue (GT) composed of connective tissue, inflammatory cells, and capillaries (black arrowhead). (Magnifications: ×100 and ×200 for (**E**) and (**F**), respectively.) (**G**–**I**) (Group III—CAP Treatment, 5 s): The burn site showed minimal healing with partial epithelialization at the wound edges (**G**). Histology indicated partial epidermal regeneration (black arrow). The dermis (DR) showed disorganized connective tissue with edema (red arrow), scattered small blood and lymphatic vessels (black arrowhead and curved arrow), and slit-like ectatic vessels beneath a partially eroded epithelium (red star). (Magnifications: ×100 and ×200 for (**H**) and (**I**), respectively.) (**J**–**L**) (Group IV—CAP Treatment, 10 s): There was a marked reduction in wound size (**J**). Histology showed epidermal regeneration (EPI) (black arrow), more organized dermal connective tissue, and the presence of blood and lymphatic vessels (black arrowhead and curved arrow), large ectatic vessels (red star), and newly formed hair follicles (thick curved arrow and yellow arrowhead). (Magnifications: ×100 and ×200 for (**K**) and (**L**), respectively.).

Gross and histological features of acute FTSBs: The skins of the backs were the sites of the acute FTSBs. There was complete damage to the epidermis, dermis, subcutis, and underlying muscle layer. A summary of these findings is shown in Figure 1A–C and Figure 2D–F.

CAP enhances wound healing and promotes full skin regeneration: In Group I—Control, the shaved back skin of the guinea pig appeared normal. Histology showed orthokeratotic stratified squamous epithelium (Keratin: red arrowhead) in the epidermis (EPI), with hair follicles (HF) and sebaceous glands (SG) in the dermis (DR), among loose (papillary) and dense (reticular) collagen fibers (Figure 2A–C. Magnifications: B—×100; C—×200).

In Group II—Untreated acute FTSBs, grossly, the burn site was extensive and covered by crusts (D). Histological analysis revealed complete skin destruction, replaced by granulation tissue (GT) composed of connective tissue, inflammatory cells, and capillaries (black arrowhead). (Figure 2D–F. Magnifications: ×100 and ×200 for E and F, respectively.)

In Group III—CAP Treatment, 5 s, the burn site showed minimal healing with partial epithelialization at the wound edges (G). Histology indicated partial epidermal regeneration (black arrow). The dermis (DR) showed disorganized connective tissue with edema (red arrow), scattered small blood and lymphatic vessels (black arrowhead and curved arrow), and slit-like ectatic vessels beneath a partially eroded epithelium (red star). (Figure 2G–I. Magnifications: ×100 and ×200 for H and I, respectively.)

In Group IV—CAP Treatment, 10 s, there was a marked reduction in wound size with most of the surface replaced by hairless skin (J). Histology showed complete epidermal regeneration (EPI), more organized dermal connective tissue, and the presence of blood and lymphatic vessels (black arrowhead and curved arrow), large ectatic vessels (red star), and newly formed hair follicles (thick curved arrow and yellow arrowhead). (Figure 2J–L. Magnifications: ×100 and ×200 for K and L, respectively.)

**Figure 3:**
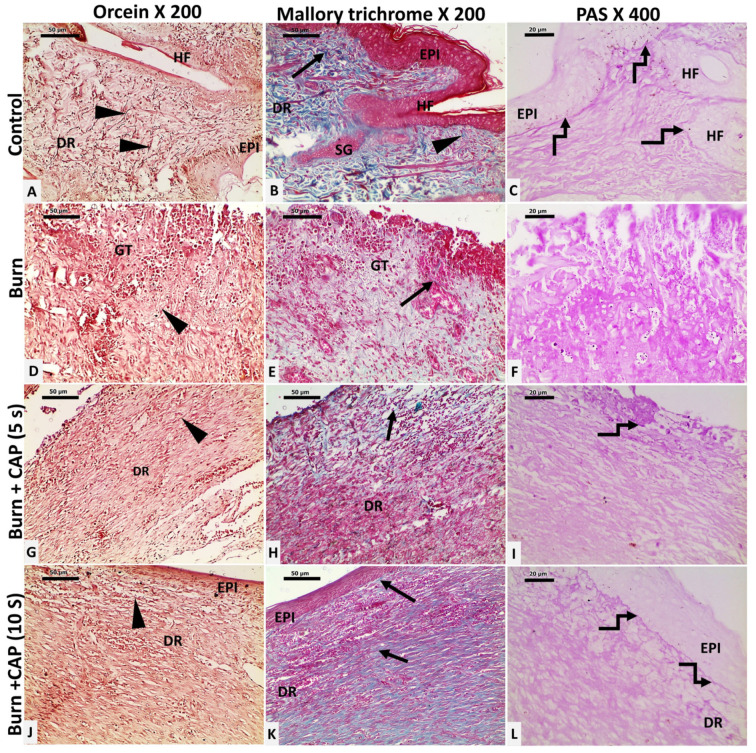
Changes in collagen and elastic fibers in guinea pig skin after acute FTSBs. (**A**–**C**) (Group I—Control): Elastic fibers appeared as slender, branching structures (black arrowhead, Orcein stain). Collagen appeared as fine fibers in the papillary dermis and thick, blue-stained wavy bundles in the reticular dermis (black arrow, Masson’s Trichrome). The basement membrane (BM) appeared as a magenta–red line beneath basal keratinocytes and hair follicles (curved arrow, PAS stain). (**D**–**F**) (Group II—Untreated acute FTSBs): Elastic (black arrowhead, Orcein) and collagen fibers (black arrow, Masson’s Trichrome) were sparse. The PAS-positive BM was absent (**F**, PAS). (**G**–**I**) (Group III—CAP, 5 s): Elastic fibers increased (**G**, black arrowhead, Orcein). Collagen fibers were slightly increased (**H**, black arrow, Masson’s Trichrome). The BM appeared patchy as PAS-positive magenta–red segments (**I**, curved arrow, PAS). (**J**–**L**) (Group IV—CAP, 10 s): Elastic fibers were abundant (black arrowhead, Orcein). Collagen fibers were significantly increased (black arrow, Masson’s Trichrome). The BM was restored as a continuous PAS-positive magenta–red line (curved arrow, PAS). Magnifications: ×200 (Orcein and Masson’s Trichrome), ×400 (PAS).

CAP stimulates basement membrane reformation, hair follicle neogenesis, and collagen and elastic fiber deposition: In Group I—Control, elastic fibers appeared as brown, slender, branching structures (black arrowhead, Orcein stain). Collagen was seen as fine fibers in the papillary dermis and thick, blue-stained wavy bundles in the reticular dermis (black arrow, Masson’s Trichrome). The basement membrane (BM) appeared as a magenta–red line beneath basal keratinocytes and hair follicles (curved arrow, PAS stain) (Figure 3A–C).

In Group II—Untreated acute FTSBs, elastic (black arrowhead, Orcein) and collagen fibers (black arrow, Masson’s Trichrome) were sparse within granulation tissue. The PAS-positive BM was absent beneath basal keratinocytes and hair follicle cells (F, PAS) (Figure 3D–F).

In Group III—CAP, 5 s, elastic fibers increased moderately (G, black arrowhead, Orcein). Collagen fibers were slightly increased, loosely arranged, and disorganized (H, black arrow, Masson’s Trichrome). The BM appeared patchy as PAS-positive magenta–red segments beneath the epidermis and hair follicles (I, curved arrow, PAS) (Figure 3G–I).

In Group IV—CAP, 10 s, elastic fibers were abundant (black arrowhead, Orcein). Collagen fibers were significantly increased, well-organized, and densely packed (black arrow, Masson’s Trichrome). The BM was restored as a continuous PAS-positive magenta–red line under the basal keratinocytes and hair follicles (curved arrow, PAS). Magnifications: ×200 (Orcein and Masson’s Trichrome), ×400 (PAS) (Figure 3J–L).

CAP significantly enhances wound closure and collagen fiber deposition: The area percentage of collagen fibers and wound closure were significantly higher in Group IV as compared to Group II or III (*p* < 0.05). (Table 3) and Figure 7. A summary of the progression in the wound size at different time points is shown in Table 4.

**Figure 4:**
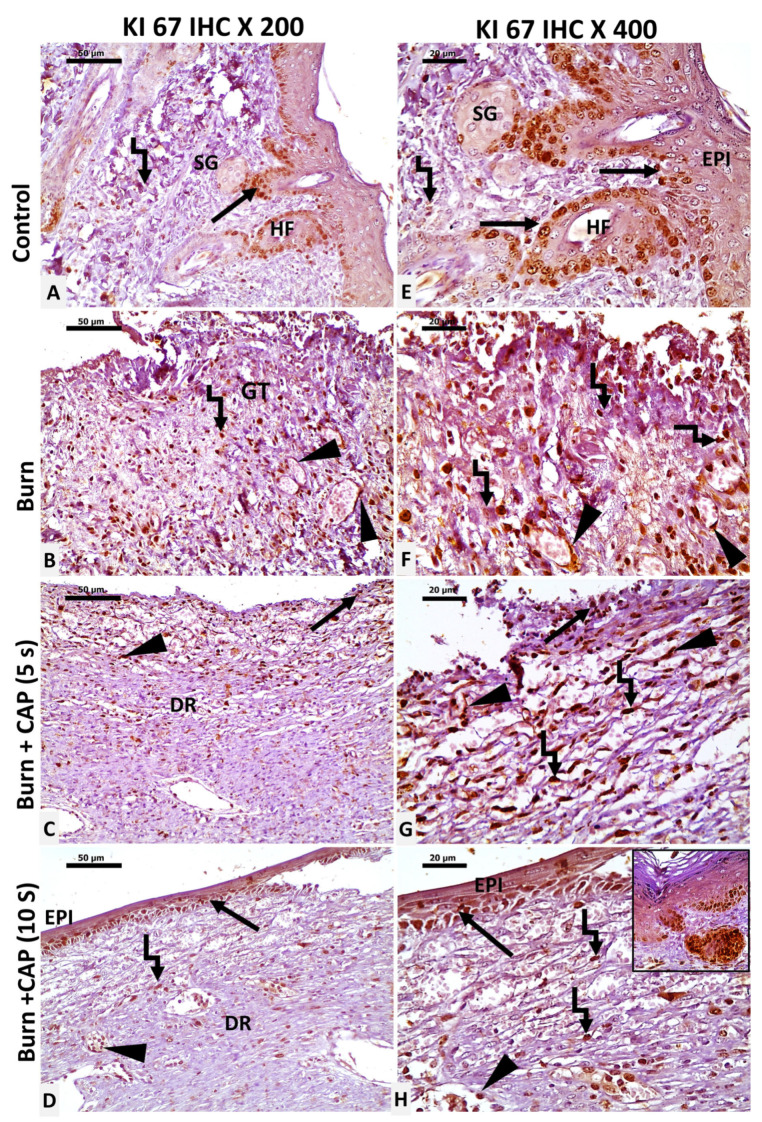
Ki-67 expression as a marker of cellular proliferation in guinea pig skin. (**A**,**E**) (Group I—Control): Ki-67 staining in basal cell keratinocytes of the epidermis and hair follicles (HF, black arrow), sebaceous glands, and scattered dermal connective tissue cells (angled arrow). (**B**,**F**) (Group II—Untreated acute FTSBs): Ki-67 positivity was observed in fibroblasts, vascular endothelial cells (black arrowhead), and inflammatory cells within granulation tissue (angled arrow). (**C**,**G**) (Group III—CAP, 5 s): Limited Ki-67 staining in basal cell keratinocytes, vascular endothelial cells (black arrowhead), and a few dermal connective tissue cells (angled arrow) and partial epidermal regeneration (black arrow). (**D**,**H**) (Group IV—CAP, 10 s): Ki-67 staining in the basal keratinocytes at the wound edges (black arrow), keratinocytes of newly formed hair follicles (inset), vascular endothelial cells (black arrowhead), and fibroblasts (angled arrow). Magnifications: ×200 (**A**–**D**) and ×400 (**E**–**H**); scale bars: 50 µm and 20 µm, respectively.

CAP increases Ki-67 proliferation index: In Group I—Control, Ki-67 nuclear staining was evident in basal keratinocytes of the epidermis and hair follicles (HF, black arrow), sebaceous glands, and scattered dermal connective tissue cells (angled arrow) (Figure 4A,E). In Group II—Untreated acute FTSBs, Ki-67 positivity was observed in fibroblasts, vascular endothelial cells (black arrowhead), and inflammatory cells within granulation tissue (angled arrow) (Figure 4B,F).

In Group III—CAP, 5 s, limited Ki-67 staining was detected in basal keratinocytes, vascular endothelial cells (black arrowhead), and a few dermal connective tissue cells (angled arrow) (Figure 4C,G). In Group IV—CAP, 10 s, Ki-67 staining was noted in basal keratinocytes at the wound edges (black arrow), keratinocytes of newly formed hair follicles (inset), vascular endothelial cells, and fibroblasts (angled arrow). Magnifications: ×200 (A–D) and ×400 (E–H); scale bars: 50 µm and 20 µm, respectively (Figure 4D,H). The Ki-67 proliferation indices were significantly higher in Group IV as compared to Group II or III (*p* < 0.05) (Table 5 and Figure 4 and Figure 7).

**Figure 5:**
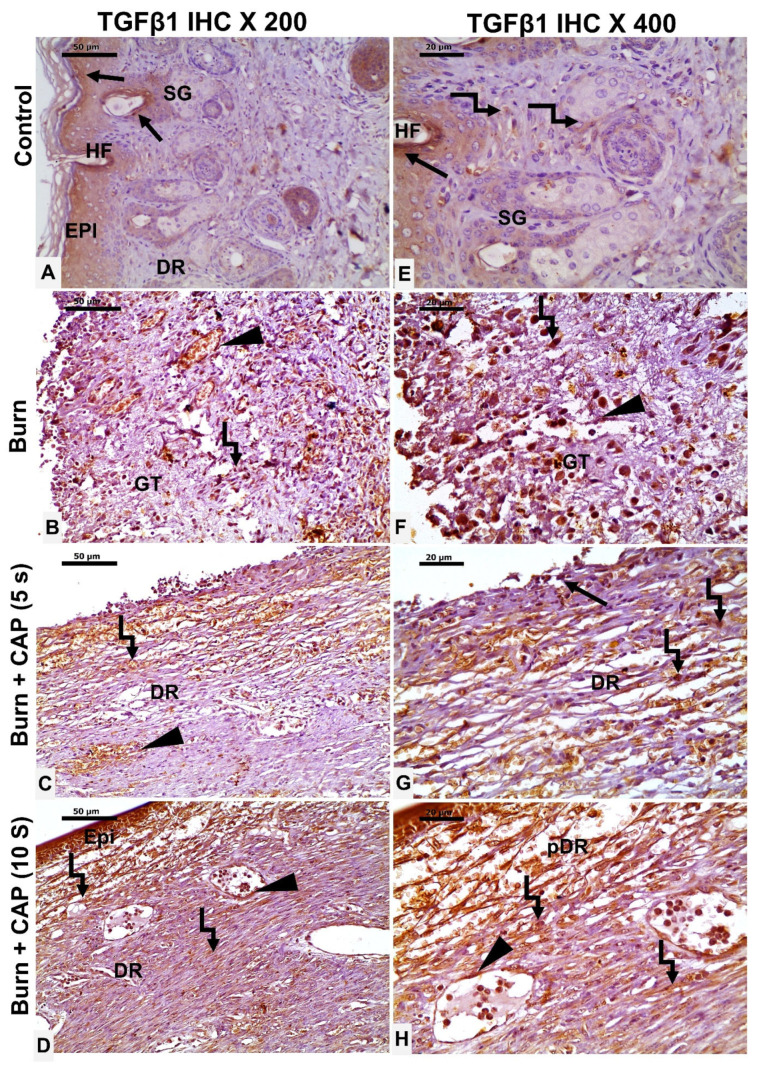
TGF-β1 expression in guinea pig skin post-acute FTSBs. (**A**,**E**) (Group I—Control): Occasional TGF-β1-positive epidermal keratinocytes (EPI), hair follicle cells (HF, black arrow), and fibroblasts (angled arrow) were observed. (**B**,**F**) (Group II—Untreated acute FTSBs): Few TGF-β1-positive fibroblasts (angled arrow), vascular endothelial cells (black arrowhead), and connective tissue cells within granulation tissue (GT). (**C**,**G**) (Group III—CAP, 5 s): Some TGF-β1-positive epidermal keratinocytes (black arrow), dermal fibroblasts (angled arrow), and vascular endothelial cells (black arrowhead) were present. (**D**,**H**) (Group IV—CAP, 10 s): Several TGF-β1-positive epidermal keratinocytes (EPI), dermal fibroblasts (angled arrow), and vascular endothelial cells (black arrowhead) were observed. Magnifications: ×200 (**A**–**D**), ×400 (**E**–**H**); scale bars: 50 µm and 20 µm, respectively.

CAP upregulates TGF-β1 and CTGF protein expression: In Group I—Control, occasional TGF-β1-positive epidermal keratinocytes (EPI), hair follicle cells (HF, black arrow), and fibroblasts (angled arrow) were observed (Figure 5A,E). In Group II—Untreated acute FTSBs, few TGF-β1-positive fibroblasts (angled arrow), vascular endothelial cells (black arrowhead), and connective tissue cells were detected within granulation tissue (GT) (Figure 5B,F).

In Group III—CAP, 5 s, some TGF-β1-positive epidermal keratinocytes (black arrow), dermal fibroblasts (angled arrow), and vascular endothelial cells (black arrowhead) were present (Figure 5C,G). In Group IV—CAP, 10 s, several TGF-β1-positive epidermal keratinocytes (EPI), dermal fibroblasts (angled arrow), and vascular endothelial cells (black arrowhead) were observed. Magnifications: ×200 (A–D), ×400 (E–H); scale bars: 50 µm and 20 µm, respectively (Figure 5D,H).

**Figure 6:**
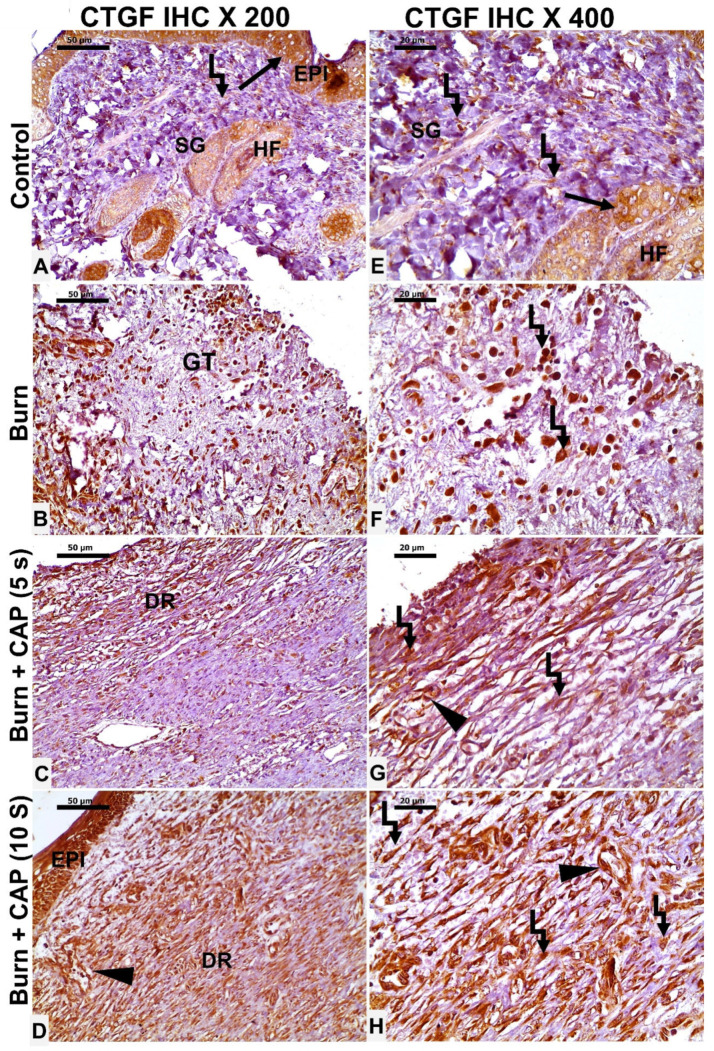
CTGF expression in guinea pig skin following acute FTSBs. (**A**,**E**) (Group I—Control): Occasional CTGF-positive epidermal keratinocytes (EPI), hair follicle cells (HF, black arrow), and fibroblasts (angled arrow) were seen. (**B**,**F**) (Group II—Untreated acute FTSBs): A few CTGF-positive fibroblasts (angled arrow), and other connective tissue cells within granulation tissue (GT). (**C**,**G**) (Group III—CAP, 5 s): CTGF expression in some epidermal keratinocytes, dermal fibroblasts (angled arrow), and vascular endothelial cells (black arrowhead). (**D**,**H**) (Group IV—CAP, 10 s): Several CTGF-positive epidermal keratinocytes (EPI), fibroblasts (angled arrow), and vascular endothelial cells (black arrowhead) were present. Magnifications: ×200 (**A**–**D**), ×400 (**E**–**H**); scale bars: 50 µm and 20 µm, respectively.

In Group I—Control, occasional CTGF-positive epidermal keratinocytes (EPI), hair follicle cells (HF, black arrow), and fibroblasts (angled arrow) were seen (Figure 6A,E). In Group II—Untreated acute FTSBs, a few CTGF-positive fibroblasts (angled arrow), vascular endothelial cells (black arrowhead), and other connective tissue cells were noted within granulation tissue (GT) (Figure 6B,F).

In Group III—CAP, 5 s, CTGF expression was detected in some epidermal keratinocytes, dermal fibroblasts (angled arrow), and vascular endothelial cells (black arrowhead) (Figure 6C,G). In Group IV—CAP, 10 s, several CTGF-positive epidermal keratinocytes (EPI), fibroblasts (angled arrow), and vascular endothelial cells (black arrowhead) were present. Magnifications: ×200 (A–D), ×400 (E–H); scale bars: 50 µm and 20 µm, respectively (Figure 6D,H).

**Figure 7:**
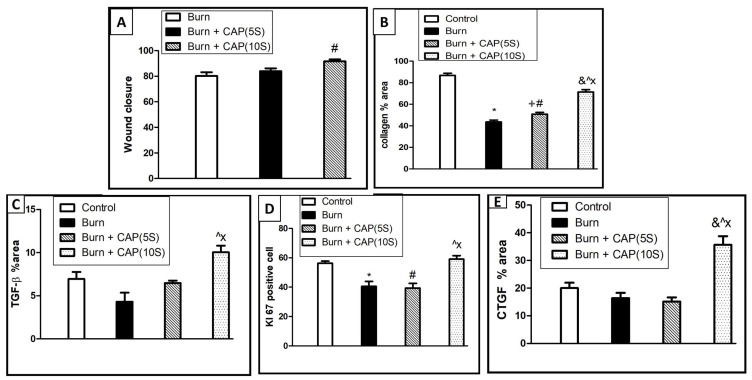
Statistical analysis of wound closure (**A**), collagen area percentage (**B**), and protein immunohistochemistry expression of anti-TGF-β1 (**C**), Ki-67 (**D**), and CTGF (**E**) in guinea pig skin. This comparative statistical analysis was designed as: Control vs. Burn; Control vs. Burn + CAP (5 s); Control vs. Burn + CAP (10 s); Burn vs. Burn+ CAP (5 s); Burn vs. Burn + CAP (10 s); Burn + CAP (5 s) vs. Burn + CAP (10 s). *: Control vs. Burn (non-significant); #: Control vs. Burn + CAP (5 s) (non-significant); &: Control vs. Burn + CAP (10 s) (non-significant); +: Burn vs. Burn + CAP (5 s) (non-significant); Λ: Burn vs. Burn + CAP (10 s) (significant *p* ≤ 0.05); X: Burn + CAP (5 s) vs. Burn + CAP (10 s) (significant *p* ≤ 0.05).

Statistical analysis of wound closure, collagen area percentage, and protein expression of TGF-β1, Ki-67, and CTGF in guinea pig skin is shown in Figure 7. The following comparisons were included: Control vs. Burn; Control vs. Burn + CAP (5 s); Control vs. Burn + CAP (10 s); Burn vs. Burn + CAP (5 s); Burn vs. Burn + CAP (10 s); and Burn + CAP (5 s) vs. Burn + CAP (10 s). (Magnification: ×400) (Figure 7). The TGF-β1 and CTGF protein expression values were significantly higher in Group IV as compared to Group II or III (*p* < 0.05) (Table 5 and Figure 5, Figure 6 and Figure 7).

## 4. Discussion

Thermal burn wounds and infected wounds differ mechanistically. Thermal burns damage the epidermal and dermal barriers, increasing susceptibility to microbial colonization, while infected wounds involve invasion by antibiotic-resistant microorganisms, leading to tissue damage, chronic inflammation, and impaired healing. CAP has shown promise as a therapeutic agent due to its antimicrobial and healing properties. Its antimicrobial effects stem from reactive oxygen and nitrogen species, UV photons, and charged particles. Its healing benefits are linked to enhanced cell regeneration, angiogenesis, and extracellular matrix remodeling [7,8]. By creating a sterile environment, CAP accelerates wound healing, highlighting its clinical potential in burn treatment [6,35]. Our study reports the following important observations.

CAP enhances wound healing and promotes full skin regeneration: The histological changes associated with acute FTSBs identified in our research align with findings from previous studies [36,37]. Consistent with the observations made by Lau et al., we found re-epithelialization at the wound margins of acute FTSBs. The changes included the regeneration of keratinocytes originating from the basal layer, the reformation of the basement membrane, and the emergence of newly formed hair follicles. These histological features likely reflect a coordinated biological response by proliferating cells to restore the integrity of the damaged tissue.

Isbary et al. reported significant amelioration of infection in chronic ulcerative wounds following the application of CAP. Their study demonstrated that a two-minute treatment with CAP effectively reduced microbial load, thereby promoting the healing process of chronic wounds [38]. Initially, CAP was used in wound management primarily for its antimicrobial properties. Subsequent research has revealed its broader therapeutic potential in wound healing [39]. CAP has been shown to enhance wound healing through various biological processes, including the improvement of cutaneous microcirculation and stimulating the proliferation of critical cell types, including keratinocytes, fibroblasts, and histiocytes [39].

CAP stimulates basement membrane reformation, hair follicle neogenesis, and collagen and elastic fiber deposition: Numerous investigations have demonstrated the critical role of collagen in accelerating the healing process of skin burns. The histological alterations observed following CAP treatment in our study are consistent with findings from earlier research [40,41]. In diabetic rat models, the reparative properties of curcumin-loaded nanofibers, utilizing gum tragacanth, were assessed, revealing that these mats not only elevated collagen levels but also facilitated wound repair [42,43]. Similarly, Mai et al. developed a hydrogel-based nano-delivery system with antibacterial properties. The system significantly enhanced wound healing by reducing inflammation, promoting collagen deposition, and accelerating epithelialization [44].

In a comparative study, Abramo et al. utilized autologous skin grafts to evaluate the healing effects of a heterologous collagen matrix sponge, derived from deer skin, against those of cotton cloth in third-degree burn wounds. The collagen sponge resulted in a significant increase in the density of endothelial cells, polymorphonuclear leukocytes, and fibroblasts. These changes occurred alongside enhanced capillary ingrowth and faster granulation tissue formation [40,41,45]. However, the potential for prolonged CAP application to induce fibrotic conditions that disrupt homeostasis warrants further research. Future research should focus on characterizing the type of newly formed collagen in relation to the expression of TGF-β1 and CTGF [46,47].

CAP increases the Ki-67 proliferation index: In burn wound healing studies, Ki-67 immunostaining (labeling index) quantifies proliferating cells, including keratinocytes, endothelial cells, and dermal fibroblasts. Assessing the Ki-67 index over time provides insight into the spatial and temporal progression of wound healing and enables evaluation of therapeutic interventions such as CAP [48,49]. Omran et al. (2024) evaluated the Ki-67 proliferation index during mucosal ulcer healing in male Wistar albino rats. Rats were assigned to three groups: normal (no ulcer), control (untreated ulcers), and treatment (ulcers treated with fenugreek leaf oil). Sacrificed at days 3 and 7, the treated group showed significantly higher Ki-67 indices, correlating with accelerated ulcer healing [49].

Durgun et al. (2023) investigated the therapeutic effects of Hesperidin on alkaline esophageal burns in Wistar albino rats. Burns were induced via oral gavage of NaOH, and rats were divided into three groups: a control group (no burns, intraperitoneal NaCl), a burn group (burn + intraperitoneal NaCl), and a treatment group (burn + intraperitoneal Hesperidin). Histological analysis showed epithelial and muscle layer degeneration in the burn group, which was reversed by Hesperidin treatment, promoting tissue regeneration. Ki-67 labeling was negative in the control group, elevated in the burn group, and reduced in the Hesperidin-treated group [48].

CAP upregulates TGF-β1 and CTGF protein expression: The study revealed an upregulation of TGF-β1 and CTGF protein expression after the application of CAP in cases of acute FTSBs. This increase was correlated with a heightened Ki-67 proliferation index, suggesting a link between these proteins and cellular proliferation during the healing process [11,45,50,51,52]. These findings support the role of TGF-β1 and CTGF as key mediators in tissue repair and fibrosis development [9].

In a related study, Henshaw et al. examined the positive effects of topical CTGF application on diabetic wounds in a rat model, where the treatment of full-thickness skin wounds with 1 μg of CTGF resulted in a faster healing rate, attributed to enhanced collagen IV deposition [53]. Their parallel clinical investigation in humans indicated that elevated levels of CTGF in post-debridement diabetic wound fluid were positively associated with the healing rate of foot ulcers. These findings suggested that CTGF may facilitate the pro-fibrotic actions of TGF-β1 during the wound healing process [24,53,54,55].

Previous studies have shown that cold atmospheric plasma (CAP) positively influences wound healing [24,56]. However, it remains unclear whether this effect is primarily due to electrical or chemical stress influencing biological processes and plasma–cell interactions. Several investigations suggest that CAP enhances wound healing through interactions between plasma-generated reactive species and cells, promoting cell migration and proliferation, as well as enhancing antioxidant and anti-inflammatory responses [57,58,59,60].

Hydrogen peroxide (H_2_O_2_) has been shown to support wound healing by increasing antioxidant activity [61], while nitric oxide (NO) promotes angiogenesis and re-epithelialization [62]. Short-lived reactive oxygen and nitrogen species (RONS)—including O, O_3_, •OH, ^1^O_2_^−^, O_2_•^−^, NO•, and ONOO^−^—act as strong oxidizers of fatty acids. This oxidative activity increases membrane permeability, which may facilitate RONS entry into cells. Peroxynitrite (ONOO^−^) can decompose into •OH and NO_2_• radicals, which react with protein tyrosine residues to form irreversible modifications such as 3-nitrotyrosine, leading to protein inactivation and lipid peroxidation [60].

This study has certain limitations that should be acknowledged. To gain a more comprehensive understanding of granulation tissue, inflammatory cell infiltration, and vascular changes, it is recommended to complement H&E staining with immunohistochemical analyses using specific markers such as CD68, CD34, smooth muscle actin, CD45, CD20, CD3, CD138, CD31, and D2-40. Additionally, assessing the Ki-67 labeling index in basal keratinocytes would benefit from concurrent staining with markers like CK19 or CK15 to ensure precise evaluation of cellular proliferation [63,64].

In summary, this study presents the immunomorphological observations obtained from the application of CAP in managing acute FTSBs. While TGF-β1 and CTGF play crucial roles in the skin’s wound healing process, they are also implicated in the development of fibrosis. TGF- β1 and CTGF serve as pivotal regulators in the initiation of fibrotic responses, with TGF-β stimulating fibroblasts to produce and contract extracellular matrix proteins [22,47,65,66]. Conversely, CTGF acts as a significant downstream effector of TGF-β. Given these findings, future research could benefit from investigating how variations in the levels of these proteins post-treatment may influence the formation of fibrotic tissue [9,65].

Furthermore, it would be worthwhile for future studies to elucidate the molecular mechanisms that drive the upregulation of TGF-β1 and CTGF, as well as to explore the therapeutic implications that may arise from these investigations.

## Figures and Tables

**Table 1:** Antibodies, reagents, and kits used in the study.

Reagents	Catalog Number	Sources
Anti-Transforming growth factor-Beta 1(TGF-β1) rabbit pAb	(Cat. No.; A15103) (Dilution of 1:200)	AB clonal, (Wuhan, China)
Anti-connective tissue growth factor (CTGF) rabbit pAb	(Cat. No.: E-AB-12339) (Dilution of 1:50)	Elab Science Biotechnology Inc., Houston, TX, United States
Anti-Ki-67 rabbit pAb antibody	(Cat. No.: GB111499), (Dilution of 1:300)	Service bio-Technology Co., Ltd., Wuhan, China
Normal goat serum	(Cat. No.: 5425) (Dilution of 10%)	Cell signaling technology, Inc., Danvers, MA, United States
Pro taqs^®^ 2 step detection goat anti-mouse/rabbit HRP with a peroxidase block and DAB chromogen, Quartet	(Cat. No DSK-211-015)	Schichauweg, (Berlin, Germany)

Note: Group II received no treatment.

**Table 2:** Animal group assignments.

Groups	Design of the Group
Group I	Healthy animals, no acute FTSBs (n = 6)
Group II	Animals suffering from acute FTSBs receiving no treatment (n = 6)
Group III	Animals suffering from acute FTSBs receiving topical CAP treatment for 5 s per day for 21 days (n = 6)
Group IV	Animals suffering from acute FTSBs receiving topical CAP treatment for 10 s per day for 21 days (n = 6)

Note: Group II received no treatment.

**Table 3:** Effects of CAP on wound closure size and collagen fiber area (%), presented as mean ± SD.

Groups	Size of the Wound Closure	Collagen Percentage Area
Group I	Non wounded	86.12% ± 6.152
Group II	81.36 ± 10.17	43.56% ± 5.784
Group III (5 s)	84.7 ± 7.11	50.83% ± 5.442
Group IV (10 s)	91.67 ± 6.087	71.36% ± 7.153

Note: Group II received no treatment.

**Table 4:** Wound size progression over time.

Groups	Size of the Wound Closure (7 Days)	Size of the Wound Closure (14 Days)	Size of the Wound Closure (21 Days)
Group I	Non wounded	Non wounded	Non wounded
Group II	33.1 ± 6.91	44.84 ± 4.037	81.36 ± 10.17
Group III (5 s)	32.93 ± 6.234	58.3 ± 3.8	84.7 ± 7.11
Group IV (10 s)	34.01 ± 6	71.48 ± 4.544	91.67 ± 6.087

Note: Group II received no treatment.

**Table 5:** Effects of CAP on TGF-β1 and CTGF expression (% area) and Ki-67 proliferation index, presented as mean ± SD.

Groups	Size of the Wound Closure	Collagen Percentage Area	TGF-β1 %Area	CTGF Positive Cell	Ki-67 Positive Cell
Groups	Size of the wound closure	Collagen percentage area	TGF-β1	CTGF	Ki-67
Group I	Non wounded	86.12% ± 6.152	6.947 ± 2.438	20 ± 5.451	56.25 ± 4.132
Group II	81.36 ± 10.17	43.56% ± 5.784 (* *p* < 0.05)	4.301 ± 3.797	16.38 ± 5.397	40.5 ± 9.577 (* *p* < 0.05)
Group III (5 s)	84.7 ± 7.11	50.83% ± 5.442 (# + *p* < 0.05)	6.471 ± 1.047 (*p* < 0.05)	15.13 ± 4.357	39.38 ± 8.975 (# *p* < 0.05)
Group IV (10 s)	91.67 ± 6.087 (@ *p* < 0.05)	71.36 % ± 7.153 (&@X *p* < 0.05)	10.04 ± 2.632 (@X *p* < 0.05)	35.63 ± 8.911 (&@X *p* < 0.05)	59 ± 6.928 (@X *p* < 0.05)

Note: Group II received no treatment. The absence of the mark indicates that the values are not significant (statistical analysis was performed using GraphPad Prism version 8.0.2 [263]). *: Control vs. Burn. #: Control vs. Burn + CAP (5 s). &: Control vs. Burn + CAP (10 s). +: Burn vs. Burn + CAP (5 s). @: Burn vs. Burn + CAP (10 s). X: Burn + CAP (5 s) vs. Burn + CAP (10 s).

## Data Availability

The original contributions presented in this study are included in the article. Further inquiries can be directed to the corresponding author.

## Abbreviations

AFTSBs	Acute full-thickness skin burns
TGF-β1	Transforming growth factor-beta1
CTGF	Connective tissue growth factor
